# Twin Reversed Arterial Perfusion Sequence Diagnosed Late in the Third Trimester: A Case Report and Literature Review

**DOI:** 10.1002/ccr3.70052

**Published:** 2025-01-16

**Authors:** Tafese Dejene, Abdi Kebede, Getahun Fetensa, Delayehu Bekele, Telila Mesfin, Kelil Hussen

**Affiliations:** ^1^ College of Medicine and Health Sciences Dire Dawa University Dire Dawa Ethiopia; ^2^ Dilchora Referal Hospital Dire Dawa Ethiopia; ^3^ Department of Health Behavior and Society, Faculty of Public Health, Institute of Health Jimma University Jimma Ethiopia; ^4^ Department of Gynecology and Obstetrics Saint Paul's Hospital Millennium Medical College Addis Ababa Ethiopia; ^5^ School of Medicine Goba General Hospital, Madda Walabu University Goba Oromia Ethiopia; ^6^ Jimma Medical Center, Department of Gynecology and Obstetrics Jimma University Jimma Ethiopia

**Keywords:** conservative management, Monochorionic diamniotic, pump twin, twin arterial perfusion sequence, ultrasound scan

## Abstract

The twin reversed arterial perfusion (TRAP) sequence is a rare complication associated with monochorionic twins. It is characterized by blood flow from the umbilical artery of the normal (pump) twin to the umbilical artery of the abnormal (acardiac) twin via artery‐to‐artery anastomosis. This condition is associated with 100% mortality in the acardiac twin and a high rate of perinatal morbidity and mortality in the pump twin, primarily due to intrauterine hypoxic injury, heart failure, and prematurity. Following delivery, the surviving pump twin may experience adverse neurodevelopmental outcomes and heart failure, necessitating ongoing follow‐up care. The goal of managing pregnancies complicated by the TRAP sequence is to deliver a healthy, near‐term pump twin through early detection, timely intervention, and continuous follow‐up. However, in low‐resource settings, such as the case presented here, the condition may progress undiagnosed into the third trimester due to a lack of experienced physicians and/or obstetric ultrasound scans. This case report serves as an entry point for a comprehensive review of the literature on management options for the TRAP sequence, specifically focusing on factors to consider when managing patients conservatively in resource‐limited environments or in cases that are referred or diagnosed late.

## Graphical abstract

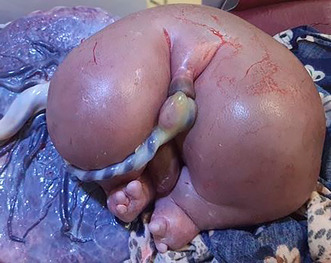

## 1 Introduction

A twin reversed arterial perfusion (TRAP) sequence is a unique, rare, and severe complication of multiple monochorionic pregnancies and is characterized by one normal fetus (pump twin) and another anomalous twin with an absent or rudimentary heart (acardiac twin) and a variable degree of deficient development of the head and upper limbs. This condition occurs in 1:9500–11,000 pregnancies and in nearly 2.6% of monochorionic twin pregnancies, with approximately 75% in diamniotic and 25% in monoamniotic twin pairs [1]. The acardiac twin receives poorly oxygenated blood, which is extracted by the acardiac twin's part of the abdomen and lower extremity, resulting in further oxygen‐depleted blood reaching the upper part of the body and eventually in varying degrees of abnormal development of the heart, head, and upper extremity [2]. The mortality rate is 100% for acardiac twins. Pump twins are at risk of high‐output heart failure, preterm delivery, and poor perinatal outcomes [3]. Although diagnosing the TRAP sequence in early pregnancy is very important for a good outcome in pump twins, the condition might progress to the third trimester undiagnosed in developing countries, such as in our case, where experienced physicians and/or obstetric ultrasound scans are not immediately available. This paper presents a TRAP sequence diagnosed late in the third trimester, delivered by cesarean section, with the survival of the pump twin. The course and outcome of this case report and literature review will support the conservative management of properly selected cases of the TRAP sequence.

## 2 Case History/Examination

A 25‐year‐old Ethiopian, primigravida woman with no significant past medical history, presented to Dilchora Referral Hospital with a referral paper from Melka Qaro Health Center. She was referred from the health center at her 9 months of amenorrhea with a diagnosis of third trimester plus rule out twin pregnancy. She received antenatal care at the health center and was given two doses of tetanus toxoid vaccination, supplemented with iron sulfate, but underwent no ultrasound scanning during pregnancy because ultrasound was not available there.

## 3 Methods (Differential Diagnosis, Investigations and Treatment)

**FIGURE 1:**
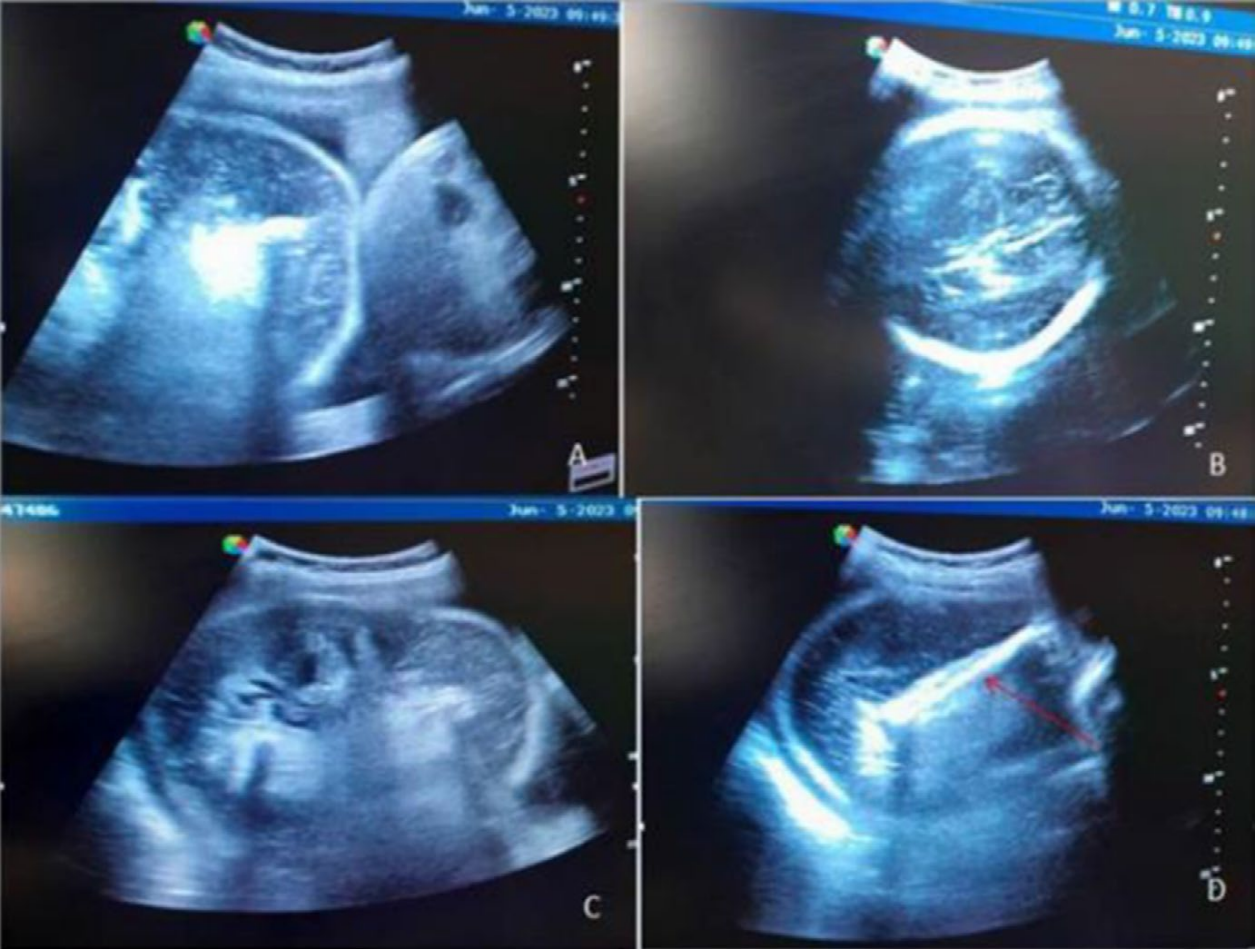
Ultrasound of features of TRAPS. (A) Pump twin at the left side of the uterine cavity and acardiac twin at the right upper side of the uterine cavity. (B) Head of pump twin. (C) The acardiac fetus—large cystic‐like tissue without visualization of cardiac activity, head, or bilateral upper limbs. (D) Femur bone of acardiac twin (red arrow).

At the hospital, obstetric ultrasonography was performed and revealed the following (Figure 1): an intrauterine monochorionic diamniotic twin pregnancy with fundal anterior placenta with one normal fetus and one acardiac fetus. The normal fetus was on the left side of the uterine cavity. It was noted in breech presentation, with grossly normal fetal morphology. Biometry revealed a gestational age (GA) of 39 weeks with an estimated fetal weight of 3750 g and no signs of hydrops. The acardiac fetus was at the right upper side of the uterine cavity. It was composed of large cystic‐like tissue with no visualization of cardiac activity, the head, or bilateral upper limbs. Both lower limbs were visualized with the femur length corresponding to 30 weeks of gestation. These ultrasound findings were consistent with the TRAP sequence in a monochorionic diamniotic twin pregnancy.

**FIGURE 2:**
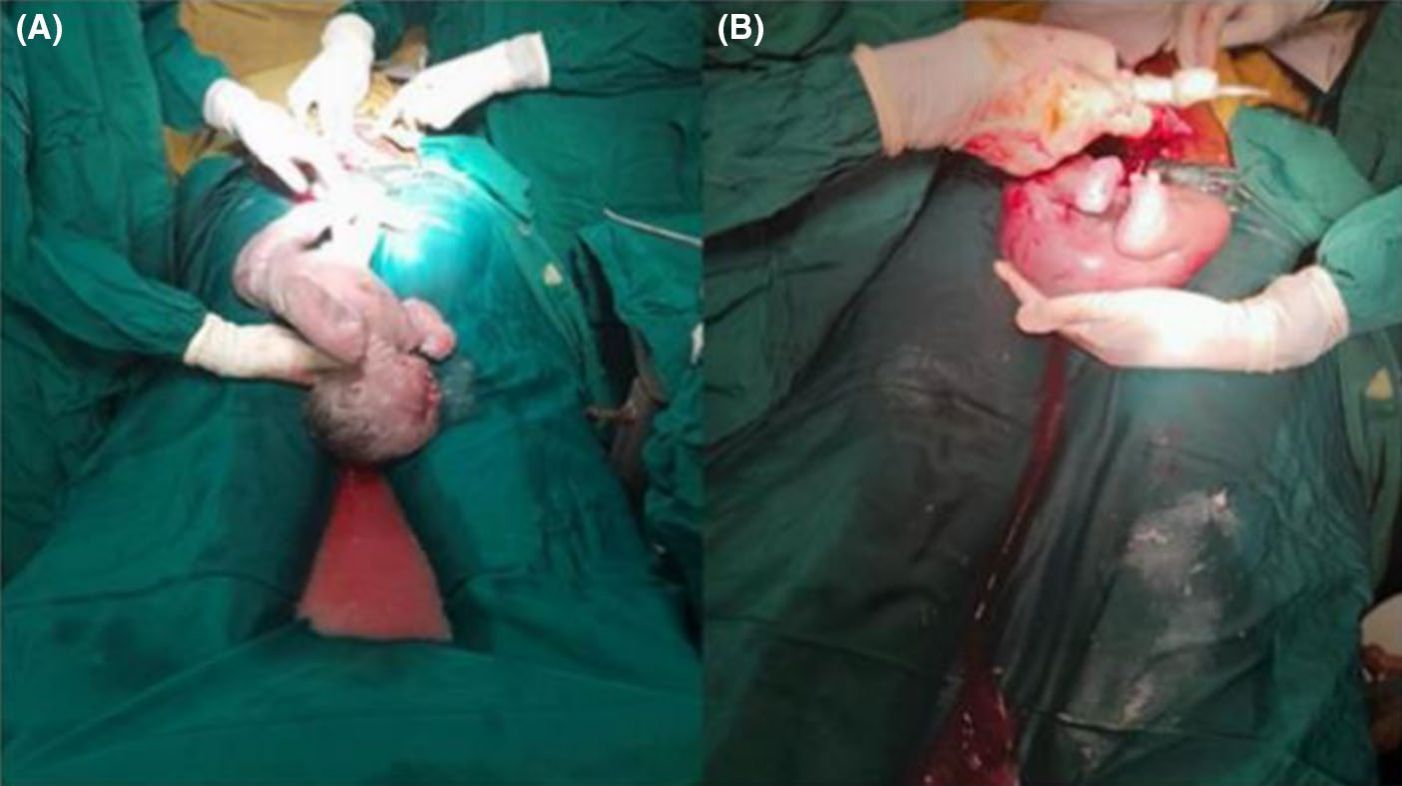
Cesarean delivery of twins with TRAPS. (A) An apparently normal pump twin, and (B) An abnormal acardiac baby.

**FIGURE 3:**
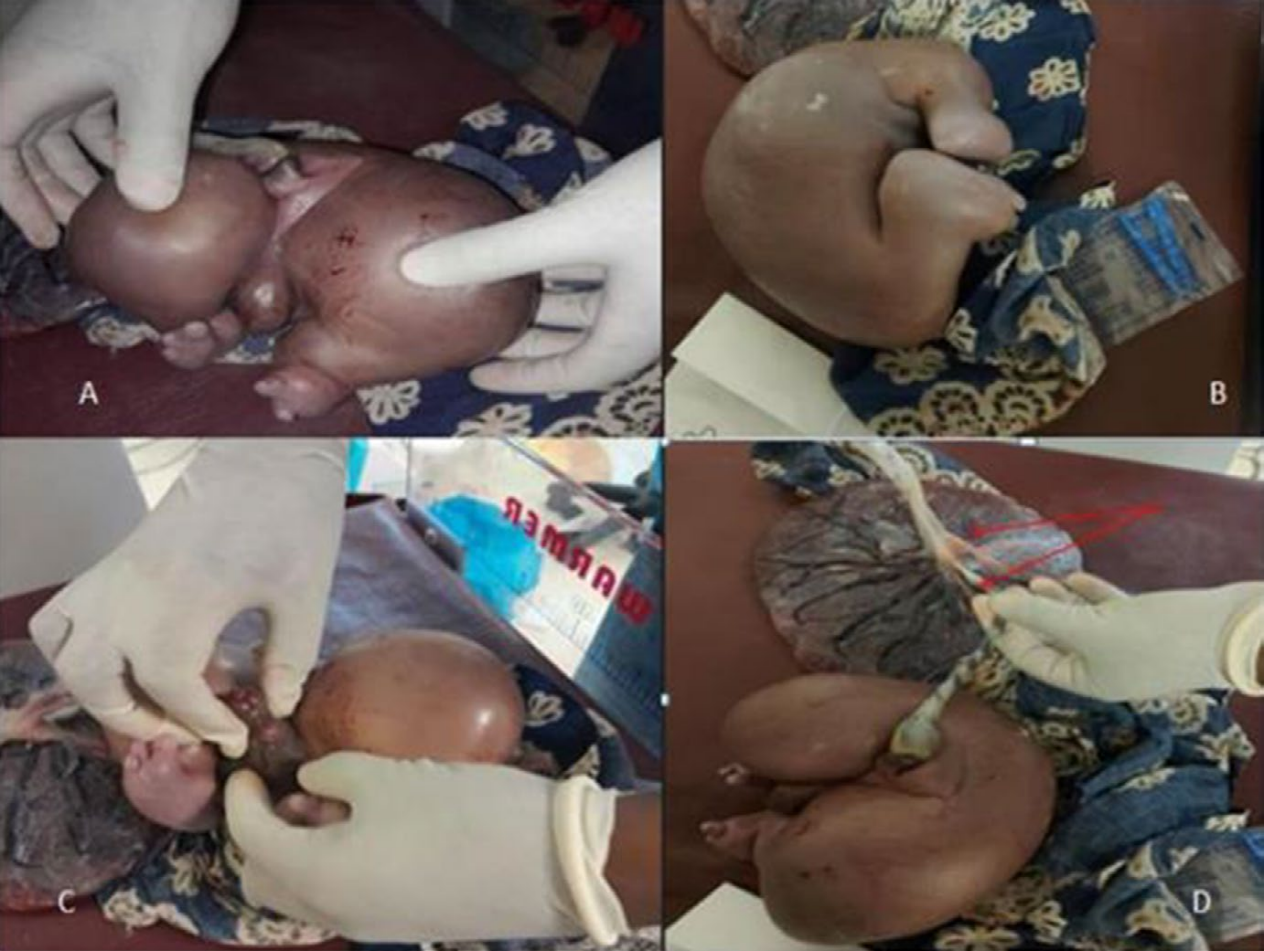
Features of the acardiac twin after delivery. (A, B) The acardiac newborn had lower limbs with three toes on the right foot and two toes on the left foot but no head or upper extremities. (C) Poorly developed male external genitalia. (D) Fetal surface of a single placenta with a thin dividing membrane (shown with arrow).

The patient was counseled, and an elective transverse lower uterine segment cesarean section was performed under spinal anesthesia for an indication of a twin pregnancy with a TRAP sequence and breech presentation. She delivered a male neonate who had no congenital anomalies, with a birth weight of 3640 g (Figure 2A). The Apgar scores were eight and nine in the first and fifth minutes, respectively. Then, an acardiac fetus weighing 1300 g was delivered (Figures 2B and 3A–C). The skin color and body temperature of the acardiac twin were normal, but there was no visible movement. The head and upper extremities were absent. The external genitals were male but poorly developed. The right foot had three toes, and the left foot had two toes. The placenta was single with a thin dividing membrane, suggesting a monochorionic diamniotic twin pregnancy (Figure 3D). It weighed 650 g. The healthy neonate was transferred to a neonatal intensive care unit (NICU) for echocardiography evaluation on the first day of life, which revealed no detectable cardiac anomaly, and returned to the mother.

## 4 Outcome and Follow Up

The neonate was thriving on breast milk during the postpartum period. The mother was sent home 3 days after delivery with her neonate. The mother disappeared from follow‐up, and it was difficult to know the child's condition after discharge.

## 5 Discussion

This case report describes a pregnant woman with a TRAP sequence diagnosed in the late third trimester, resulting in the delivery of a healthy, normal twin along with an acardiac cotwin.

The TRAP sequence in twin pregnancy is a rare congenital anomaly characterized by the formation of a malformed fetus with an absent or rudimentary (but nonfunctional) heart. It seems to be a particular form of Twin‐To‐Twin Transfusion syndrome (TTTS), in which the donor is the normal fetus, which supplies blood for itself, and the acardiac fetus is regarded as the recipient. Without prompt early detection, follow‐up, and treatment, the mortality rate for donor twins has been reported to be as high as 50%–70%, while that for acardiac twins is 100% [4, 5, 6].

Prenatal diagnosis of the TRAP sequence can be made using transabdominal ultrasound at the end of the first trimester [7, 8]. The diagnosis can be established at an early gestational age (11–12 weeks) through transvaginal ultrasound and 3D ultrasound [9, 10]. An acardiac twin should be suspected when an ultrasound examination identifies one of the fetuses with evident morphologic abnormalities in monochorionic twins [7, 8]. An acardiac twin may be detected sonographically, showing fetal growth and movement, without definite differentiation of the head and trunk [7, 8]. It is commonly associated with maldeveloped upper and lower extremities in the absence of a morphologically normal heart [8]. Sometimes, irregular cardiac activity can be detected as a result of a rudimentary heart beating or retrograde pulsation from the pumping twin. Furthermore, there may be subcutaneous edema and fluid collection in the anomalous twin, and gross differences in biometrical measurements of the acardiac and pump twins, particularly in abdominal circumference, may also be detected [8, 11]. Doppler studies are also very helpful for diagnosing TRAP sequences and evaluating fetal safety in utero. They may show pathognomonic features demonstrating reversed arterial flow in the umbilical artery from the pump twin to the acardiac twin [12]. Most acardiac twins present with umbilical artery malformations such as single umbilical artery and three‐vessel umbilical cord thrombosis [13], which can be detected with Doppler color flow. Arterial blood is pumped from the pump twin to the acardiac twin, which can affect the hemodynamic status and fetal cardiac function. If left untreated, in the majority of cases, the pump twin develops high‐output heart failure, including cardiomegaly, pericardial effusion, and tricuspid regurgitation with polyhydramnios [12]. Therefore, echocardiography is mandatory for the assessment and follow‐up of cardiac function in pump twins [12]. Intrauterine fetal demise and intra‐amniotic or placental tumors such as placental teratomas in monochorionic twins could resemble an acardiac fetus, and precise evaluation via ultrasound could aid in differentiation [14]. An acardiac fetus shows maintained growth and movement at ultrasound follow‐up, and Doppler assessment of a fetus suspected of being acardiac in utero reveals persistent intrafetal blood flow signals; however, Doppler assessment of a monochorionic twin suspected of being demised in utero should reveal the absence of blood flow [15]. Rare differential diagnoses such as intra‐amniotic or placental tumors can be ruled out by precise evaluation of spinal development and umbilical cord attachment [16]. In our patient, the condition was diagnosed at 9 months of amenorrhea after a referral from a rural health center with suspicion of twin pregnancy. The TRAP sequence was diagnosed following ultrasound findings of intrauterine monochorionic diamniotic twin pregnancy with a grossly normal fetus on the left side of the uterine cavity noted in breech presentation and a large cystic‐like anomalous fetus having lower limbs, with no visualization of cardiac activity, head, or bilateral upper limbs on the right upper side of the uterine cavity.

The acardiac fetus is clinically divided into two broad categories: the holoacardius and the hemiacardius [17]. The holoacardius is the absence of cardiac structure development. The hemiacardius is the presence of some rudimentary cardiac structures. The acardiac fetus is also further classified into four distinct types based on morphology: [5, 18, 19]

— Acardius acephalus: is the most common type, in 60%–75% of cases, and is characterized by a well‐developed pelvis and lower extremities and an absence of the head, thoracic organs, and upper extremities. Our patient is in this group.
— Acardiac anceps: in approximately 20% of cases, body shape and extremities developed with rudimentary development of the head and face.
— Acardiac acormu: is quite rare and accounts for approximately 10% of all cases; it is characterized by the presence of only the head, while the body (if present) is a shriveled mass.
— Acardiac amorphous: accounting for approximately 5% of all cases, characterized by the absence of a recognizable fetal structure, and the fetus appears to be a shapeless mass.
— Acardiac myelacephalus: characterized by a partially developed head with identifiable upper extremities and/or some nervous tissue.

This morphological description has no prognostic value and does not provide information about the best management option.

The acardiac twin is incompatible with life after delivery owing to its parasitic reliance on the blood supply from the pump twin [5, 15]. The perinatal mortality rate of pump twins is approximately 50%–70% [4, 20, 21]. Pump twins have high perinatal mortality owing to increased hemodynamic stress placed on their heart to perfuse the acardiac twin [22]. Therefore, it is necessary to effectively predict the occurrence of the TRAP sequence and screen for potential prognostic factors that may threaten the pump twin. One of the prognostic factors is the size of the acardiac twin. After the diagnosis of the TRAP sequence, the weight of the acardiac twin and pump twin should be calculated because the twin weight ratio is an essential prognostic factor for the outcome of a TRAP pregnancy [8]. However, the exact weight of the acardiac twin cannot be calculated using standard formulas based on ultrasound biometry, such as Hadlock's formula, because of the lack of well‐developed anatomic structures. The weight of the acardiac twin can be estimated from its longest length by using the following formula: weight (grams) = 1.2 × (longest length in centimeter)^2^ – (1.7 × longest length in centimeter), and based on the weight ratio, the pregnancy outcome is favorable if the weight ratio of the acardiac twin to pump twin in TRAP is less than 70% and worse if the ratio is greater than 70% [21, 23]. The measurement of the acardiac fetal weight may be difficult to obtain, and in that case, the ratio of the abdominal circumference (AC) of the acardiac twin to that of the pump twin can be measured to predict the prognosis of the pump twin, and a ratio ≥ 50% is considered a poor prognostic factor [24]. When we extrapolate this concept in our case, the estimated weight of our anomalous twin based on a maximum length of 41 cm was approximately 1579.9 g, and using Hadlock's formula, the estimated weight of the normal twin was 3750 g. The weight ratio of anomalous to normal twins was 42.1%. Hence, based on Moore's equation, our TRAP sequence pregnancy had a good prognosis for the pump twin, which was supported by the post‐delivery outcome of our baby, which was in good health. The AC of our normal fetus was 37 cm, but it was difficult to measure the AC of the anomalous fetus because its abdomen was not identified. Hence, it was not possible to predict the prognosis of the normal fetus by the AC ratio.

The other indicators of poor prognosis for pump twins are the following: [15, 25, 26]

— Polyhydramnios (the deepest vertical pocket > 8 cm).
— Cardiac failure in the pump twin, as evidenced by cardiomegaly, pericardial effusion, and tricuspid regurgitation detected by fetal echocardiography, and Doppler sonography revealing diastolic waves inversed on the umbilical artery and flow inversed on the ductus venosum.
— Fetal hydrops in the pump twin.

The outcome of the pump twin can be affected by at least three mechanisms: [24, 27]

— Preterm premature membrane rupture (PPROM), preterm labor, and preterm delivery caused by uterine over distension due to polyhydramnios and larger acardiac twins.
— Congestive heart failure (CHF) of the pump twin, caused by increased cardiac work due to increased blood flow.
— Hypoxia and intrauterine growth restriction (IUGR) of the pump twin, caused by poorly oxygenated blood that returns to the pump twin through the vascular anastomosis.
— If the TRAP sequence occurs in a monochorionic monoamniotic pregnancy, another poor prognostic factor for the pump twin is the risk of cord entanglement.

In our case, during ultrasound scanning, there were no identified poor prognostic factors that could affect the pump twin.

The optimal management of pregnancy with a TRAP sequence is controversial because it is rare, and there is ongoing discussion regarding correct management, conservative or intervention, and the exact time of intervention. The goal of management of the TRAP sequence is to preserve the survival of the pump twin and reach the term for delivery. The TRAP sequence can be treated in different ways depending on the condition of the pumping twin [9, 18]. Conservative management is recommended for pregnant mothers with a TRAP sequence in which the acardiac twin is small in size and there is no sign of cardiovascular compromise in the pump twin. Conservative management is aimed at close monitoring for early detection and in utero management of complications in pump twins. It includes regular follow‐up with ultrasonography, Doppler flow studies and fetal echocardiography for the early detection of complications such as polyhydramnios, cardiac dysfunction, abnormal Doppler‐flow patterns, and hydrops and for in utero treatment of these complications. It includes amniocentesis or medical treatment of polyhydramnios and tocolytic treatment of preterm labor to reduce the risk of preterm delivery to improve the survival prognosis of the pump twin and inotropic drug treatment to treat heart failure and support the cardiac performance of the pump twin [28, 29, 30]. This conservative management approach incurs a minor risk and seldom improves the outcome of the pump twin by preventing procedures‐related fetal and maternal complications [18, 31, 32]. The outcome of conservative management, when the weight of the acardiac twin is < 50% of that of the pump twin, has been reported to be favorable in 88% of cases [18, 33]. However, other studies reported high pregnancy loss rates with conservative management, with a TRAP sequence diagnosed in the first trimester and pregnancy loss rates of 83%–100%, with all losses occurring at ≤ 16 weeks of gestation [9, 34, 35, 36]. Therefore, pregnant women whose acardiac twins weight is > 50% of that of pump twins or whose TRAP sequence involves monoamniotic twins have a poorer prognosis and should be treated by invasive intervention [36]. Multiple treatment options that aim to discontinue the perfusion of the acardiac twin have been introduced. These interventions target the umbilical cord vessels, the vascular anastomoses on the placental surface, or the intrafetal vessels [37, 38]. These procedures include hysterotomy with selective delivery of the acardiac twin, interruption of vascular communications by endoscopic laser coagulation or ligation of the acardiac twin's umbilical cord, ultrasound‐guided embolization of the acardiac twin's umbilical artery with absolute alcohol, the use of thrombogenic coils, and intrafetal ablation of the acardiac twin with radiofrequency ablation [20, 32, 38, 39, 40]. However, these procedures may lead to many complications, such as premature membrane rupture, preterm labor, placental abruption, intrauterine infection, fetal hemorrhage, fetal death, and maternal pulmonary edema [15, 31, 32]. These management options are not immediately available in the institutions of developing countries, including our set‐up. They are found at a small number of institutions in the developed world, with none having clearly demonstrated superiority. There are also no clear guidelines on the appropriate timing of intervention to prevent the death of the pump twin [11]. That said, the best management method for pregnant mothers with a TRAP sequence has not yet been determined and is controversial because most patients with a TRAP sequence can be diagnosed as early as the first trimester, where it is impossible to predict the outcome. Although various sonographic findings suggest that impending pump twins deteriorate, most of these findings are not predictive of perinatal outcomes.

Findings from case reports indicate that children who survived after conservative management for TRAP sequence exhibited poor neurodevelopmental outcomes during long‐term follow‐up [22, 41]. In contrast, those who survived after treatment with minimally invasive modalities for vascular anastomosis demonstrated favorable long‐term neurodevelopmental outcomes, particularly when the diagnosis was established at an early gestational age and treated promptly [10, 42, 43]. After delivery, the neonate should be monitored for neurodevelopmental complications and signs of heart failure, with ongoing ultrasounds [44]. In our patient, the condition was diagnosed via an ultrasound conducted in the late third trimester. During the scan, no poor prognostic factors were identified. The pump twin was in good health at the time of discharge. However, the patient subsequently discontinued follow‐up, making it challenging to evaluate the long‐term outcome of the child after delivery. Our case is compared with other conservatively managed case reports published previously in Table 1.

**TABLE 1:** Review of the literature on outcomes of twin pregnancies complicated by twin reversed arterial perfusion sequence managed conservatively.

References	GA at the time of diagnosis of TRAP sequence (in weeks)	Complications identified during pregnancy	Outcome
Aoyagi et al., 2019 [40]	13	— Blood flow to the acardiac twin disappeared spontaneously at 18 weeks — Pump twin developed IUGR	Delivered vaginally female alive neonate at 39 weeks and 1 day weighing 1891 g and acardius acephalus
Enesia Ziki et al., 2019 [41]	26	— Absent diastolic flow in the umbilical artery of the pump twin — Pump twin developed IUGR	Delivered by cesarean section at 34 weeks female alive weighing 1370 g and 3000 g acardius acephalus
Blickstein, 2007 [42]	18–19	Polyhydramnios, preterm labor	Preterm delivery at 24 weeks delivered, pump twin died on the second day because of respiratory failure
Kanagal et al., 2013 [43]	34	—	Delivered vaginally female alive 1700 g and 900 g acardiac acephalus
Kanagal et al., 2013 [43]	24	Polyhydramnios, preeclampsia	Delivered by cesarean section at 36 weeks male alive weighing 2000 g and 750 g acardius myelocephalus
Kanagal et al., 2013 [43]	24	—	Delivered by cesarean section female alive neonate weighing 2100 g and 700 g acardius amorphous
Quaas & Markfeld‐Erol, 2021 [44]	First trimester	— IUGR and preterm labor — Steady growth of theacardiac twin	Delivered by cesarean section at 24 weeks alive male pump twin weighing 510 g and 1200 g male acardius anceps
Quaas & Markfeld‐Erol, 2021 [44]	13	— IUGR, oligohydramnios — Spontaneous regression of the acardiac mass	Delivered vaginally at 35 weeks pump baby weighing 2200 g and 86 g degenerated parasitic twin
Quaas & Markfeld‐Erol, 2021 [44]	First trimester	—	Delivered by cesarean section at 35 weeks alive pump twin 2765 g and 1340 g acardius amorphous
Pepe et al., 2015 [18]	25	Polyhydramnios, preterm labor, preterm premature rupture of membrane	Delivered by cesarean section at 36 weeks alive female weighing 2550 g and 1300 g acardius acephalus
Behura et al., 2020 [45]	30	Polyhydramnios	Delivered vaginally at 36 weeks and 6 days alive male neonate weighing 2430 g and acardius aneps
Kapote & Mohite, 2022 [28]	22	Polyhydramnios	Delivered by cesarean section at 37 weeks alive female neonate weighing 2900 g and 500 g acardius acephalus
Yıldırım, 2019 [46]	31	Polyhydramnios	Delivered by cesarean section at 34 weeks twin A weighing 2010 g, twin B weighing 2150 g and 500 g acardius
Martimucci et al., 2018 [47]	28	Polyhydramnios, left ventricular hypertrophy, tricuspid and mitral valve regurgitation	Delivered by cesarean section at 32 weeks and 6 days alive neonate weighing 2489 g and 995 g acardius
Martimucci et al., 2018 [47]	28	Mild cardiomegaly and mild tricuspid regurgitation	Delivered by cesarean section at 29 weeks and 2 days alive neonate weighing 1000 g initially admitted to NICU with diagnosis of a respiratory distress syndrome and discharged improved on the seventh day Acardius twin weighed 1000 g
Martimucci et al., 2018 [47]	22	Cardiac dysfunction of the pump twin was suspected and counseled for radiofrequency ablation but the patient declined	4 days later pump twin demised, induced and delivered dead pump twin weighing 452 g and 388 g acardius
Our case	39	—	Delivered by cesarean section alive male neonate weighing 3640 g and 1300 g acardius acephalus

## 6 Conclusion

When resources permit, early diagnosis of TRAP sequence and intervention, rather than a conservative approach, may be a more effective option for achieving favorable short‐ and long‐term perinatal outcomes. In settings where resources are limited or cases are diagnosed or referred late, a conservative approach may be considered after conducting a thorough risk–benefit assessment. After delivery, the neonate should be monitored for neurodevelopmental complications and signs of heart failure.

There are limited studies that have assessed the long‐term neurodevelopmental outcomes of surviving pump twins. This highlights the need for larger, multicenter studies that evaluate the long‐term neurodevelopmental outcomes of children who survived after various management options for TRAP sequence. Such research is essential to refine the current criteria for identifying ideal candidates and determining the optimal timing for fetal intervention to achieve favorable perinatal short‐ and long‐term outcomes.

## Author Contributions

**Tafese Dejene:** conceptualization, data curation, writing – original draft. **Abdi Kebede:** conceptualization, data curation, investigation. **Getahun Fetensa:** conceptualization, formal analysis, investigation. **Delayehu Bekele:** conceptualization, investigation, methodology. **Telila Mesfin:** formal analysis, supervision. **Kelil Hussen:** conceptualization, writing – review and editing.

## Ethics Statement

Ethical clearance was obtained from the institutional review board of Dire Dawa University.

## Consent

Written informed consent was obtained from the patient for the publication of this case report and accompanying images. A copy of the written consent is available for review by the editor‐in‐chief of this journal. Consent to Participate: Consent for publication was obtained.

## Conflicts of Interest

The authors declare no conflicts of interest.

## Data Availability

The authors have nothing to report.
